# Draft genome sequences of two multidrug-resistant *Enterobacter soli* strains isolated from wastewater in Spain

**DOI:** 10.1128/mra.01029-24

**Published:** 2024-11-29

**Authors:** Marta Lois, Sergio Rodríguez, Jesús L. Romalde

**Affiliations:** 1 CRETUS, Department of Microbiology and Parasitology, CIBUS-Faculty of Biology, Universidade de Santiago de Compostela, Santiago de Compostela, Spain; 2 ARCUS, Department of Microbiology and Parasitology, CIBUS-Faculty of Biology, Universidade de Santiago de Compostela, Santiago de Compostela, Spain; DOE Joint Genome Institute, Berkeley, California, USA

**Keywords:** *Enterobacter soli*, wastewater, antibiotic resistance, sulfonamides, fluoroquinolones

## Abstract

We report the draft genome sequences of two *Enterobacter soli* strains isolated from wastewater in Galicia (NW Spain). Genomic analysis revealed that both *E. soli* strains harbor multiple genes associated with resistance to sulfonamides, fluoroquinolones, β-latamics, and other drugs.

## ANNOUNCEMENT

The genus *Enterobacter* contains a variety of species with different behaviors and impacts on their environments and hosts (1). *Enterobacter soli* was initially isolated from soil in 2011 (2). Later, this species was reported as a causative agent of gastro-hepato disease in fish (3), and it was recently isolated from human blood in a patient diagnosed with acute pancreatitis and bloodstream infection (4). More recently, extended-spectrum β-lactamase producing *E. soli* strains were detected in wastewater in New Zealand (5). In our study, two antibiotic-resistant *Enterobacter soli* strains were isolated and characterized from sewage samples in Galicia (North-West Spain) in a decentralized system. Wastewater samples (2 L) were collected on October 2022, in Nigrán (42.20599062 N 8.73005254 W). Samples were serially diluted, and 100 mL of dilutions were filtered through 0.45 µm pore size nitrocellulose sterile membrane filters. Filters were placed on MacConkey Agar plates supplemented separately with ciprofloxacin (1 µg mL^−1^), trimethoprim (100 µg mL^−1^), and sulfamethoxazole (7 µg mL^−1^) and incubated for 24–48 h at 37°C. For this study, two isolates (a10 and a12), identified as *Enterobacter soli* by biochemical tests and 16S rRNA gene sequencing (6, 7), were selected for further characterization. Both strains were isolated from grey waters, strain a10 from the inflow, and strain a12 from the effluent of the treatment system. Multiresistance of these strains was confirmed in antibiogram assays on Mueller-Hinton agar using the disk diffusion method (8) under the Clinical and Laboratory Standards Institute guidelines (9) (Table 1).

**TABLE 1:** Assembly statistics for the genome sequences reported in this study

Genome	GenBankaccessionno.	SRAaccession no.	Number of reads	Avg coverage(×)	Total contig length	Number of contigs	N50	Largest contig size (nt)	G+C (%)	CDS number	Total genes	Protein-coding genes	rRNA genes	tRNA genes	Resistance to^*a*^
a10	JBDOQX000000000	SRR30160831	4,763,314	35	5,123,700	160	57,102	209,685	53.99	4,894	4,958	4,830	5	51	RL CIP AML KF S E
a12	JBDOQW000000000	SRR30160830	4,231,397	35	5,122,353	183	44,653	213,195	54.09	4,895	4,958	4,825	4	51	RL CIP AML KF W CT E

^*a*^ Results obtained by disk diffusion method. Antibiotics (µg/disk): RL, sulphamethoxazole (25); CIP, ciprofloxacin (5); AML, amoxicillin (25); KF, cephalotin (30); S, streptomycin (10); E, erythromycin (5); W, trimethoprim; CT, colistin sulfate (50).

Genomic DNA was isolated from pure cultures using a DNeasy Blood & Tissue kit (Qiagen, Mississauga, Ontario, Canada), following the manufacturer’s instructions. The DNA was sequenced at Sistemas Genómicos (SGLabs, Valencia, Spain). Sequencing libraries were prepared with the Nextera XT DNA Library Prep kit and sequenced with the MiSeq instrument (Illumina) using paired-end read technology. The quality of the raw data was checked using FASTQC v0.9.1 (www.bioinformatics.bbsrc.ac.uk/projects/fastqc) (10), and quality trimming procedures were performed using Trimmomatic version 0.39 (11). Sequences were assembled with Megahit (12). The publicly available genome was annotated using the NCBI Prokaryotic Genome Annotation Pipeline v6.7 (13). Resistance genes were evaluated using ResFinder v4.5.0. (https://cge.food.dtu.dk/services/ResFinder/) and the Comprehensive Antibiotic Resistance Database (CARD v3.2.9; https://card.mcmaster.ca/analyze/rgi), with a minimum identity of 95%. Average nucleotide identity (ANI) among the different *E. soli* genome assemblies was calculated by PYANI 0.2.11 (14) and plotted in a heatmap. Default parameters were used for all software unless otherwise specified.

**Fig 1:**
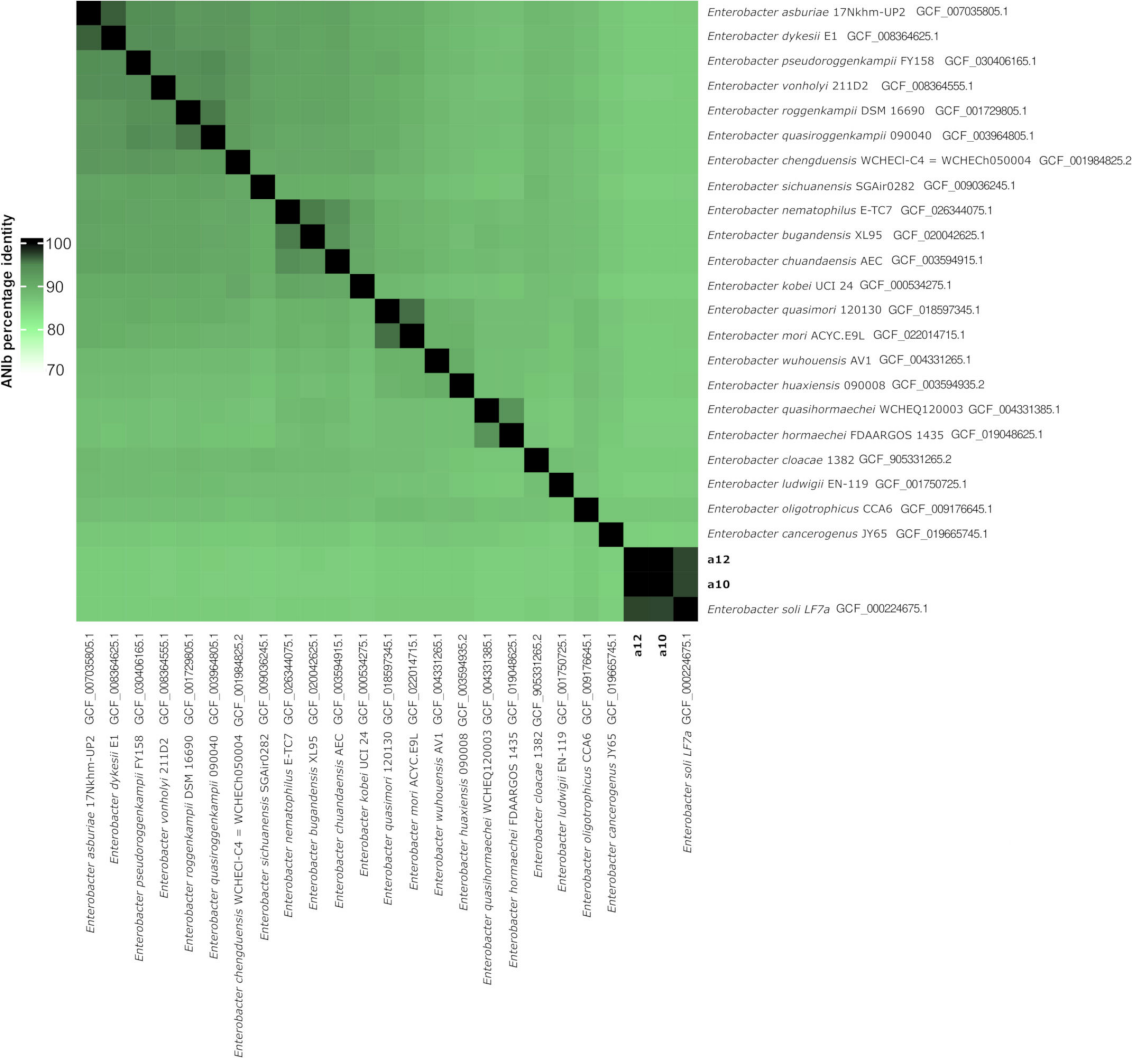
Heatmap based on percentage of average nucleotide identity for the two *Enterobacter* strains that were analyzed in this study and the type strains of the species with the genus *Enterobacter*. Values above 70% are represented in green, and values above 95% are represented in dark green and black.

In the annotated result, strains yielded an ANI of 97.35% with the type strain of *Enterobacter soli* LF7 (GCF_000224675.1) (Fig. 1). Detailed statistical metrics for each genome sequence are in Table 1. The sulfonamide resistance gene *sul2* was found in both genomes of *E. soli*. Other resistance genes to fluoroquinolones and β-lactamics (among other drugs) were found in the *E. soli* assemblies, including *adeF*, *baeR*, *baeS*, *CRP*, *emrB*, *emrR*, *H-NS*, *marA*, *rsmA*, *oqxA*, *ArnT*, *ACT-8*, *FosA2*, *vanG*, *msbA,* and *leuO*.

On the basis of these and previous results, it seems clear that the detection and environmental dissemination from wastewater of multiresistant *Enterobacter soli*, a species with demonstrated pathogenic potential for humans and animals, is worthy of investigation.

## Data Availability

The whole-genome sequencing project has been deposited at NCBI under BioProject accession number PRJNA1113845; Biosamples SAMN41469027 (a10) and SAMN41469028 (a12). Raw sequences have been deposited to Sequencing Read Archive under accession numbers SRR30160831 (a10) and SRR30160830 (a12). The assembled genome sequences were deposited at NCBI under accession numbers JBDOQX000000000 (a10) and JBDOQW000000000 (a12).
